# STUDYING AGING ABROAD: TWO INTENSIVE COURSES AT DREXEL UNIVERSITY

**DOI:** 10.1093/geroni/igz038.2668

**Published:** 2019-11-08

**Authors:** Kristine A Mulhorn, Jesse F Ballenger, Katherine F Clark, F Yoshinaga

**Affiliations:** Drexel University, Philadelphia, Pennsylvania, United States

## Abstract

The model for short-term study abroad courses---called Intensive Courses Abroad (ICA) is one where various topics can be covered under a single theme over a 10-to-14 day travel timeframe. Students participate in pre-departure assignments, a daily schedule in the host country with meaningful visits followed by group discussions, and a final presentation upon return to the U.S. In both examples presented, the topic is aging. In one, there is a visit to Japan, focusing on aging, technology and culture. Students consider design and lifestyle implications of an aging society. The course includes visits to a geriatric rehabilitation facility and to a residential facility that employs various robots and other technology. Students explore a remote community and its design challenges for an aging society. Participation in a community survey to address a question they plan to pursue for their final project, such as the role of technology in society, how we understand the aging process, and how culture defines aging. In the second course, global aging frames discussions on the way Chile is adopting mechanisms to address chronic conditions associated with aging, including dementia. In the course, students learn about the social and political context. Students observe various ways Chile has adopted innovative approaches to address dementia care and various community-level interventions. Students will reflect in journals regularly and give a presentation about the ways the country is facing the challenges of an aging society where more than 30% of the population is projected to be over 60 by 2050.

